# Type and physical intensity of occupations at pulmonary TB diagnosis

**DOI:** 10.5588/ijtldopen.24.0323

**Published:** 2025-01-01

**Authors:** M. Saroufim, C. Geric, A. Majidulla, A. Abjani, G. Tavaziva, S. Saeed, A.J. Khan, F. Ahmad Khan

**Affiliations:** ^1^ Respiratory Epidemiology & Clinical Research Unit, Centre for Outcomes Research & Evaluation, Research Institute of the McGill University Health Centre, Montreal, QC, Canada;; ^2^ Interactive Research and Development (IRD) Pakistan, Karachi, Pakistan;; ^3^ The Indus Hospital, Karachi, Pakistan;; ^4^ IRD Global, Singapore;; ^5^ McGill International TB Centre, McGill University, Montreal, QC, Canada.

**Keywords:** employment, physical activity, post-tuberculosis lung disease

## Abstract

**BACKGROUND:**

Pulmonary TB (PTB) predominantly affects individuals of working age. We sought to characterise the occupations of people newly diagnosed with PTB in Karachi, Pakistan, by type and physical intensity.

**DESIGN/METHODS:**

We did a secondary analysis of data from a study evaluating the diagnostic accuracy of artificial intelligence-based chest X-ray (CXR) analysis software, where individuals had been evaluated for active PTB using sputum cultures and had provided information on occupation. We used an accelerometer-validated US National Health and Nutrition Examination Survey-based job categorisation to assign physical activity levels to participant-reported occupations as High, Intermediate, or Low.

**RESULTS:**

Among 272 participants with microbiologically confirmed PTB (women: 130/272, 48%; median age: 29 years, IQR 22–45), 78% (211/272) had smear-positive disease, and 96% (260/272) had data on occupation. Unemployment was common (women: 70/122, 57%; men: 23/138, 17%). Most women reporting an occupation were homemakers (21/52, 40%), and 54% (28/52) had an intermediate- or a high physical activity occupation. Among men reporting an occupation, 35% (40/115) were labourers, and 79% (91/115) had an intermediate- or high-physical activity occupation.

**CONCLUSION:**

The majority of individuals with PTB were in their working age, had extensive disease, and had intermediate or high physical activity occupations, suggesting economic vulnerability due to physical impairment.

## RÉSUMÉ

**CONTEXTE:**

La TB pulmonaire (PTB) touche principalement les personnes en âge de travailler. Nous avons cherché à caractériser les professions des personnes nouvellement diagnostiquées avec PTB à Karachi, au Pakistan, par type et intensité physique.

**CONCEPTION/MÉTHODES:**

Nous avons effectué une analyse secondaire des données d'une étude évaluant la précision diagnostique d'un logiciel d'analyse par radiographie thoracique (CXR) basé sur l'intelligence artificielle, où des individus avaient été évalués pour une PTB active à l'aide de cultures d'expectorations et avaient fourni des informations sur la profession. Nous avons utilisé une catégorisation des emplois basée sur l'enquête américaine sur la santé et la nutrition pour attribuer des niveaux d'activité physique aux professions déclarées par les participants comme étant élevées, intermédiaires ou faibles.

**RÉSULTATS:**

Parmi 272 participants atteints de PTB microbiologiquement confirmé (femmes : 130/272, 48% ; âge médian : 29 ans, IQR 22–45), 78% (211/272) avaient une maladie à frottis positif et 96% (260/272) avaient des données sur la profession. Le chômage était fréquent (femmes : 70/122, 57% ; hommes : 23/138, 17%). La plupart des femmes ayant déclaré une profession étaient des femmes au foyer (21/52, 40%), et 54% (28/52) avaient une activité physique intermédiaire ou élevée. Parmi les hommes ayant déclaré une profession, 35% (40/115) étaient des ouvriers, et 79% (91/115) occupaient une activité physique intermédiaire ou élevée.

**CONCLUSION:**

La majorité des personnes atteintes de PTB étaient en âge de travailler, souffraient d'une maladie étendue et occupaient des professions d'activité physique intermédiaires ou élevées, ce qui suggère une vulnérabilité économique due à une déficience physique.

TB predominantly occurs in economically active age groups,^1^ and a substantial proportion of individuals with pulmonary TB are left with chronic respiratory impairment.^2–4^ Incidence and risk of TB associated with particular occupations have been well-researched (e.g. mining,^5^ healthcare,^6,7^ public transit,^8^ textile and iron and steel industries,^9^ agricultural labour, cleaning, and refuse work^10^). By contrast, there is a lack of data on the distribution of occupations held by persons with TB; however, it is possible that the occupations that place people at higher risk of TB are not the occupations most commonly held by people with TB. Furthermore, if it is common for patients with active TB to work in physically demanding occupations before or at TB diagnosis, this could have important implications for clinical interventions, social support programmes, and research to mitigate the economic consequences of post-TB lung disease. Gaining insight into the types of occupations and the level of physical activity held by people at the time of TB diagnosis could help us understand the potential magnitude of the negative impact of post-TB lung disease on loss of employment and income.

We sought to assess the types of occupations and their physical intensity among individuals newly diagnosed with pulmonary TB in a high TB burden country. To do so, we used data from a study that prospectively enrolled people being evaluated for possible active pulmonary TB in Karachi, Pakistan.^11^

## METHODS

### Study design and population

The original study sought to evaluate the diagnostic accuracy of computer-aided detection (CAD) software for analysing chest radiographs for pulmonary TB. From 10 March 2017 to 13 July 2018, we consecutively enrolled patients presenting to the Ghori TB Clinic of the Indus Hospital, Karachi, Pakistan, a private, non-profit, free-care tertiary institution.

We enrolled individuals aged ≥15 years presenting with cough, weight loss, fever, haemoptysis, or night sweats or those identified as a household contact of someone with active TB, regardless of symptom status. On the day of enrollment, participants underwent a standardised questionnaire, digital chest X-ray (CXR), height and weight measurements, random glucose testing, and had 3 sputum samples collected (2 for mycobacterial liquid culture, 1 for nucleic acid amplification testing with Xpert^®^ MTB/RIF [Cepheid, Sunnyvale, CA, USA]). We induced sputum expectoration when required, using ultrasonic nebulisation of hypertonic saline. We excluded individuals with active TB and those within 1 year of TB treatment completion. For the current study, we included all individuals diagnosed with active pulmonary TB, which we defined as at least one culture-positive for *Mycobacterium tuberculosis.*

### Variables of interest

We collected data on sex, age, self-reported prior TB, diabetes, tobacco smoking status, symptoms, body mass index, sputum smear status (acid-fast bacilli positive vs negative), and occupation. Data were collected on standardised paper forms in Urdu, verified by a supervisor, and then transcribed into an electronic database. For occupational data, participants were asked about their current or most recent occupation and up to three prior occupations. Due to substantial missing data, we do not report on prior occupation. All non-English responses to questions about occupations were translated into English by Karachi-based research staff (AA, AM). With respect to employment status, we classified individuals as either Employed, Unemployed, Retired, Student or Homemakers (used instead of ‘housewife’^12^). Three researchers (AA, AM, MS) grouped occupation responses that were variations of the same job into categories (e.g. garment factory worker and garment labour were grouped as ‘garment worker’).

To provide an overview of occupational categories in this study population that could be easily compared with other reports, we grouped occupations into sub-major categories of the Pakistan Standard Classification of Occupations (PSCO), which is derived from the International Standard Classification of Occupations (ISCO) and that does not classify students, retirees and homemakers.^13^ To assign a physical activity level to occupations, we used the accelerometer-derived classification system of Steeves et al.,^14^ which is based on the US National Health and Nutrition Examination Survey (NHANES) job categories. We used this approach because data on the physical intensity of occupations collected from Pakistan or other low-income countries were unavailable. Briefly, Steeves and co-investigators created a physical intensity classification for the 40 job categories of the 2003–2004 NHANES using accelerometry data collected in a sub-sample of participants aged 20–60 years. They calculated a physical intensity summary score based on six accelerometer-derived indicators (total activity counts/day, activity counts/minute, proportion of wear time spent in moderate-to-vigorous physical activity, lifestyle, light, and sedentary behaviour) with classifications of high, intermediate, and low physical activity corresponding to tertile of the physical intensity summary index. To apply the physical activity classification of Steeves et al., we assigned each occupation reported by our participants to one of the 40 NHANES job categories. Students were assigned Low physical activity, and homemakers were classified as Private household occupations and, therefore, assigned Intermediate physical activity. We did not classify participants who were missing data on occupation or were retired or unemployed.

### Statistical analysis

We assumed that occupation types would differ between women and men, so all analyses were stratified by self-reported gender. We used descriptive statistics to summarise demographic data, occupation, and physical intensity of occupations. When calculating percentages for categories of physical intensity, we excluded participants who were retired, unemployed or had missing occupational data. We used two approaches to explore associations between the severity of TB at diagnosis and reported occupational physical intensity at diagnosis. First, we compared sputum smear status between the three categories of occupational physical intensity, using Fisher’s exact tests to calculate p-values. Second, we used the Kruskal-Wallis test to compare the extent of chest radiographic disease between categories of occupational physical intensity using the continuous TB abnormality scores generated by two commercially available computer-aided detection software (CAD4TB v6, Delft, The Netherlands; qXR v3, qure.ai, Bengaluru, India). We used 0.05 as a cut-off for statistical significance. All statistical analyses were performed on R v4.1.2 (R Computing, Vienna, Austria).

### Ethics statement

The institutional review boards of Interactive Research and Development (Karachi, Pakistan) and the Research Institute of the McGill University Health Centre (Montreal, QC, Canada) approved the study. The original study informed consent included permission for the secondary use of data.

## RESULTS

Of the 2,311 participants enrolled in the original study, we included 272 participants diagnosed with culture-confirmed pulmonary TB, of which 48% (130/272) were female. The median age was 29 years old (interquartile range [IQR] 22–45), 13% (36/272) reported prior TB, and 78% (211/272) had sputum smear-positive disease. Among men, 78% (107/138) were employed, 17% (23/138) reported unemployment, and 6% (8/138) were students. Among women, 57% (70/122) reported unemployment, 17% (21/122) were homemakers, 14% (17/122) were employed, and 12% (14/122) were students. There were no retirees (Table 1).

**Table 1.** Characteristics of participants with PTB included in this analysis.

Variable	Overall (*n* = 272) *n* (%)	Women (*n* = 130) *n* (%)	Men (*n* = 142) *n* (%)
Age, years, median [IQR]	29 [22–45]	25 [19–35.8]	35.5 [25–51]
Prior TB	36 (13)	17 (13)	19 (13)
Smear-positive	211 (78)	94 (72)	117 (82)
Employment status			
Employed*	124 (48)	17 (14)	107 (78)
Unemployed	93 (36)	70 (57)	23 (17)
Homemaker	21 (8)	21 (17)	0 (0)
Student	22 (9)	14 (12)	8 (6)
Retired	0 (0)	0 (0)	0 (0)

* Twelve individuals with PTB (8 women and 4 men) missing information.

PTB = pulmonary TB; IQR = interquartile range.

Table 2 gives the occupational categories stratified by gender, excluding participants who reported being unemployed. The most common occupational category was homemaker among women (21/52, 40%) and labourer among men (40/115, 35%). Supplementary Table S1 (https://doi.org/10.6084/m9.figshare.27725088.v3) lists occupations and PSCO categorisations of occupations stratified by sex, excluding unemployed, homemaker and student participants. The most common PSCO category was Professionals (10/17, 59%) among women and Elementary Occupations among men (40/107, 37%). Supplementary Table S2 (https://doi.org/10.6084/m9.figshare.27725088.v3) lists each occupation reported by participants, its assigned occupational categorisation in our study, the corresponding PSCO occupational classification (Major Group), NHANES job category, and physical intensity level based on the Steeves et al. study.^14^

**Table 2.** Occupation categories stratified by sex among participants diagnosed with pulmonary TB.*

Occupation	*n* (%)
Women (*n* = 52)	
Homemaker	21 (40)
Student	14 (27)
Teacher	7 (13)
Healthcare worker	2 (4)
Housekeeper	2 (4)
Beautician	1 (2)
Company worker	1 (2)
Doctor	1 (2)
Garments worker	1 (2)
Nurse	1 (2)
Sales worker	1 (2)
Men (*n* = 115)	
Labourer	40 (35)
Sales worker	10 (9)
Student	8 (7)
Other driver	6 (5)
Garments worker	5 (4)
Security guard	5 (4)
Company worker	3 (3)
Farmer	3 (3)
Helper	3 (3)
Sales owner/supervisor	3 (3)
Teacher	3 (3)
Housekeeper	2 (2)
Mason	2 (2)
Net worker	2 (2)
Printing worker	2 (2)
Waiter	2 (2)
Army soldier	1 (1)
Butcher	1 (1)
Call centre worker	1 (1)
Cleaner	1 (1)
Clerical worker	1 (1)
Electrician	1 (1)
Furniture polisher	1 (1)
Garments owner/supervisor	1 (1)
Launderer	1 (1)
Machine operator	1 (1)
Mailman	1 (1)
Nurse	1 (1)
Painter	1 (1)
Plumber	1 (1)
Steel mill worker	1 (1)
Welder	1 (1)

* Among women, 8 had missing current occupation information and 70 were unemployed. Among men, 4 had missing current occupation information and 23 were unemployed.

Table 3 reports the physical intensity level of the reported occupations, stratified by gender, excluding unemployed individuals. Among men, 56% (64/115) of occupations were categorised as high physical intensity. Among women, 6% (3/52) were in high physical activity occupations, and 48% (25/52) had intermediate physical activity. However, if Homemakers were classified as having high physical intensity, 46% (24/52) of women would be considered to have high physical intensity occupations (Supplementary Table S3; https://doi.org/10.6084/m9.figshare.27725088.v3). Supplementary Tables S4 and S5 (https://doi.org/10.6084/m9.figshare.27725088.v3) compare the distribution of PSCO categorisations of occupations of women and men in our participants, respectively, to those of the Pakistan general population labour force (2020–2021).^15^ Among women, the most prevalent occupational categories in our participants were Professionals (59%) and Service Workers and Shop and Market Sales Workers (29%), versus Skilled Agricultural and Fishery Workers (60.5%) and Elementary Occupations (13.7%) in the general population. Among men, the most prevalent occupational categories in our participants were Elementary Occupations (37%) and Service Workers and Shop and Market Sales Workers (25%), versus Skilled Agricultural and Fishery Workers (25.2%) and Service Workers and Shop and Market Sales Workers (16%) in the general population.

**Table 3.** Physical activity of occupation at time of pulmonary TB diagnosis stratified by gender.*

Physical activity	Women (*n* = 52) *n* (%)	Men (*n* = 115) *n* (%)
High	3 (6)	64 (56)
Intermediate	25 (48)	27 (23)
Low	24 (46)	24 (21)

* Individuals with no information on current occupation or unemployed at time of diagnosis were excluded (women: *n* = 78; men: *n* = 27).

As shown in Tables 4 and 5, occupational physical activity at the time of diagnosis was not associated with smear status or the extent of radiographic disease as quantified by computer-aided detection software.

**Table 4.** Physical intensity of occupation at the time of pulmonary TB diagnosis stratified by smear status.*

Physical intensity	Smear-positive (*n* = 134) *n* (%)	Smear-negative (*n* = 33) *n* (%)
High	55 (41)	12 (36)
Intermediate	43 (32)	9 (27)
Low	36 (27)	12 (36)

* Individuals with no information on occupation or unemployed at the time of diagnosis were excluded (smear-positive with no physical activity level: *n* = 77; smear-negative with no physical activity level: *n* = 28; *P* = 0.57, Fisher’s exact test).

**Table 5.** Chest radiograph TB abnormality scores stratified by physical intensity of occupation at the time of pulmonary TB diagnosis, overall and stratified by gender.*^†^

Physical intensity of occupation	CAD4TB, median [IQR]			qXR, median [IQR]		
	Overall (*n* = 167)	Women (*n* = 52)	Men (*n* = 115)	Overall (*n* = 167)	Women (*n* = 52)	Men (*n* = 115)
High	82 [74–89]	93 [86–95]	82 [74–89]	0.91 [0.89–0.94]	0.91 [0.91–0.92]	0.91 [0.87–0.94]
Intermediate	79 [76–86]	80 [74–94]	79 [77–87]	0.92 [0.86–0.94]	0.91 [0.86–0.93]	0.92 [0.87–0.95]
Low	78 [73–84]	78 [69–87]	79 [74–82]	0.91 [0.87–0.94]	0.91 [0.85–0.94]	0.92 [0.88–0.94]

* Scores were generated using two commercially available computer-aided detection software (CAD4TB, Delft Imaging, Delft, The Netherlands; and qXR, qure.ai, Bengaluru, India).

† When comparing scores between rows of each column, all *P* values were less than 0.05 using Kruskal-Wallis test, indicating no statistically significant association between the scores and physical intensity.

## DISCUSSION

Among individuals newly diagnosed with culture-confirmed pulmonary TB in Karachi, Pakistan, the majority of participants were of working age, had advanced disease as measured by sputum-smear and CAD abnormality score, and reported occupations categorised as high or intermediate physical intensity. Homemaking was the most common occupation reported by women, while most men were labourers. For both men and women, unemployment rates were approximately three times higher than national gender-specific averages reported in Pakistan’s urban areas in 2018-2019 (men: 17% vs. 6.5%, women: 57% vs. 17.1%).^15^ The unemployment rate reported among women should be interpreted with caution, as it is possible that some women were engaged in unpaid household work.

There is a lack of data describing the distribution of different occupations among people diagnosed with TB, and there is no published data on the physical effort these occupations require. In a study undertaken in Malawi, data were not available on specific types of occupations or levels of physical activity associated with them.^16^ Furthermore, using the ISCO, Babalik et al. reported the most frequent occupational group among TB patients in a Turkish hospital was ‘Craft and Related Trades Workers’ for men and women, with elementary occupations ranking third in frequency among men and fourth among women.^17^ In both our study and that of Babalik et al.,^17^ the two most common occupations reported by women were homemaking and student. To note, women who reported being housewives were not classified as unemployed, but as homemakers. Applying the PSCO classification in our study— which is adapted from the ISCO classification—we found that ‘Elementary Occupations’ and ‘Professionals’ were the most reported job types among men and women, respectively, albeit the sample size of women eligible for a PSCO categorisation was very small (*n* = 17) because homemakers and students are not included in the PSCO. The distribution of occupational categories in our study population differed from that reported for the general population in Pakistan.^15^ These differences should be interpreted with caution, partly due to the limited sample size— particularly among women— and could be due to our study being conducted in an urban setting or a socioeconomic class distinct from the general population. Our findings could be useful for occupation-based active case-finding and emphasise the importance of considering gender when planning such interventions.

We acknowledge that our findings do not provide direct insight into why individuals may have higher rates of unemployment and sustained income loss despite being cured of TB. However, demonstrating a high frequency of occupations categorised as high or intermediate physical intensity makes it plausible that post-TB lung disease^2–4^ compromises return to such work, leading to unemployment and income loss. Hence, our findings— focused on occupation at diagnosis—are complementary to observations in Malawi that did not provide data on type or physical intensity of occupation (pre- or post-treatment) but demonstrated associations between abnormal spirometry and respiratory symptoms one-year post-TB treatment with unemployment and loss of income.^16^ Together, our studies further emphasise the importance of preventive treatment and early diagnosis of active disease to mitigate post-TB physical impairment. Additionally, it suggests that in settings similar to ours, providing job training for less physically demanding occupations could be an important intervention to lessen the economic impact of pulmonary TB.

Our study has several strengths. First, the TB diagnostic algorithm minimised the potential for misclassification of disease status. Second, the linguistic and contextual expertise of the Karachi-based study staff enabled verification and grouping of participant responses about their jobs into common categories and also enabled the application of the national standard job categorisation (PSCO), which facilitates comparison of results with studies undertaken in different locations. Third, our approach to classifying occupational physical activity was based on an accelerometry-verified classification.^14^ Fourth, to our knowledge, this is the first study seeking to describe occupation-related physical activity among people with active TB. We believe our approach could easily be applied to other programmatic settings to increase understanding of the types of occupations and their associated physical activity level held by people with active TB.

Our study also has several limitations. First, the original study had not collected data on occupational physical activity, and we could not find studies from Pakistan or other low-income countries reporting on the physical intensity of occupations; hence, we applied a US population- and occupation-derived classification method which could have led to some misclassification of physical activity levels of jobs in Pakistan. While future studies could use the World Health Organization Global Physical Activity Questionnaire,^18^ concerns have been raised about its validity against direct measures such as accelerometers.^19^ Second, we were only able to undertake a cross-sectional analysis of current occupation and TB at the time of diagnostic testing. Third, we did not have data to further explore the differences in unemployment between men and women; moreover, it is possible that we have overestimated ‘unemployment’ among women, as some may have been economically active within their households but did not consider themselves employed. Fourth, as a cross-sectional analysis with data at the time of diagnosis, we could not demonstrate a direct correlation between the physical intensity of occupation, post-TB lung disease, unemployment and income loss.

In summary, in a TB referral centre in Karachi, at the time of active TB diagnosis, the majority of men and women holding an occupation had high or intermediate physical intensity jobs and had extensive pulmonary disease; moreover, unemployment was common. Further research, in this setting and others, is needed to advance understanding of the interplay between TB disease, labour, gender, and the economic impacts of TB. In particular, prospective studies to determine if individuals with active TB employed in physically demanding jobs are at greater risk of sustained job loss and whether post-TB lung disease mediates such an association are needed.
